# Development of a Culinary Intervention (Cooking Class) for Salt Reduction in Japanese Home Cooking: Strategies and Assessment

**DOI:** 10.1016/j.focus.2024.100227

**Published:** 2024-03-13

**Authors:** Miyuki Imamoto, Toshihiko Takada, Sho Sasaki, Yoshihiro Onishi

**Affiliations:** 1 Department of Food and Human Nutrition, Faculty of Human Life Science, Notre Dome Seishin University, Okayama, Japan; 2 Research Association for Applied Dietary and Physical Therapy (ADAPT), Ashiya, Japan; 3 Department of General Medicine, Shirakawa Satellite for Teaching And Research (STAR), Fukushima Medical University, Fukushima, Japan; 4 Section of Education for Clinical Research, Kyoto University Hospital, Kyoto, Japan; 5 Center for Innovative Research for Communities and Clinical Excellence (CiRC2LE), Fukushima Medical University, Fukushima, Japan

**Keywords:** Salt reduction, consumer education, cooking class, Japanese diet, home cooking, nonrandomized interventional study

## Abstract

**Introduction:**

Culinary interventions (cooking classes) are a potential educational tool for salt reduction in the home diet, but their content has never been reported in detail. This study aimed to develop a cooking class for salt reduction, describe its rationale and structure so that other parties could replicate it, and preliminarily assess its impact on salt intake.

**Methods:**

A multidisciplinary research team developed a cooking class package to reduce salt content in the Japanese home diet. The package comprised its developmental policy, teaching methodology, a menu and recipes, and an implementation manual and aimed to allow third parties to replicate and modify the content. The team took the following step-by-step developmental approach. First, traditional home meals were modeled to create strategies contributing to a target of 2 g salt/meal. Then, educational topics were developed through these strategies, and finally, a dietitian produced menus and prepared documents for the class. The impact of the cooking class was assessed in a nonrandomized study of community residents. The outcome was differences in urinary salt excretion before and after the intervention. General linear models were used to account for the possible confounders.

**Results:**

The authors assumed 4–7 g salt/meal from analyzing typical Japanese home diets and developed 3 strategies: (1) restricting salt content in the main dish, (2) maintaining good tastes without salty dishes, and (3) balancing nutrition with low-salt dishes. On the basis of these strategies, the authors selected a total of 5 educational topics that participants could learn and apply at home: 1a, a simple and reliable technique to limit salt in a serving; 2a, excluding salty dishes; 2b, staple foods with notable flavor and aroma; 3a, flavoring without salt in side dishes; and 3b, ingredients that should be used intentionally. The team dietitian translated these educational topics into a menu and recipes for hands-on training and prepared a manual for conducting the class. The class developed using this approach was successfully overseen by a dietitian outside the research team. In the validation study, the intervention group (*n*=52) showed a greater decrease in urinary salt excretion than the control group (*n*=46), with an adjusted difference of −1.38 g (*p*=0.001).

**Conclusions:**

The authors developed a cooking class package for salt reduction so that third parties could replicate and modify the class. The significant salt reduction noted in this study warrants further studies to apply this cooking class to other populations.

## HIGHLIGHTS

• Development of a cooking class to reduce salt in Japanese home cooking.
• Strategies, menu, and recipes for salt reduction based on analysis of traditional Japanese cuisine.
• Knowledge, techniques, and taste experience of salt reduction that can be applied at home.
• A complete document for holding low-sodium cooking classes.
• An effect of 1.38 g/day salt reduction in local residents in an interventional study.

## Graphical Abstract

**Figure:**
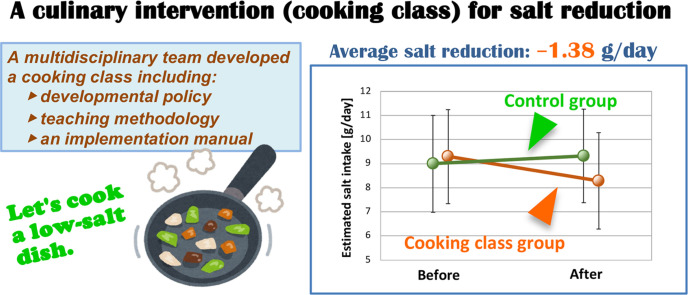

## INTRODUCTION

Salt reduction is a global health challenge. Excessive salt intake is a major cause of hypertension and the greatest dietary risk to health.^1^^,^^2^ In 2013, a 30% reduction in salt intake was one of the goals set by the WHO to reduce noncommunicable diseases.^3^ Countries around the world have been taking action based on strategies for salt reduction, including changes in the composition of processed foods through industry outreach; consumer education; clear indication in ingredient labeling; and educational interventions in schools, workplaces, and other public facilities.^4^ The sources of salt intake vary by country^5^^,^^6^; in Europe and North America, processed foods and restaurant meals account for the majority of salt intake, whereas in China and Japan, salt added at home is the largest source. In countries where home diet is the dominant source of salt, efforts to improve consumer education are important.

In Japan, the government, academic societies, and medical institutions have conducted publicity efforts, published books, held seminars, and hosted cooking classes to encourage consumers to reduce salt in their home diets.^7^, ^8^, ^9^ Home diets differ depending on the individual's environment and culture, and the sense of taste is habituated by the preferences developed through their home diet. Therefore, to successfully reduce salt intake, individuals' sense of taste and habits must first be changed. Salt is a powerful flavor accent. Effective education requires more than providing general knowledge and advice; strong guidance accompanied by experience will be required as well.^10^

Culinary interventions (cooking classes), which in general include teaching methodology, menus and recipes, and curriculums, have been deemed a reasonable approach to educating the public on such matters. Several Japanese studies have demonstrated the effects of cooking classes on salt reduction; Kitaoka et al.^11^ reported a cooking class for urban community residents in a non-RCT. They noted a salt reduction of 0.9 g/day, emphasizing the measurement of seasoning and controlling the saltiness of soups as salt-reduction techniques. Takada and colleagues^12^ reported on a cooking class for rural community residents in an RCT. The authors showed participants the amount of salt in a normal diet and had participants taste a light-flavored meal containing low salt; through these efforts, a salt reduction of 1.2 g/day was achieved by participants.

Hoffmann et al.^13^ noted that “without a complete published description of interventions, clinicians and patients cannot reliably implement interventions that are shown to be useful, and other researchers cannot replicate or build on research findings.” To establish cooking classes as an evidence-based intervention for salt reduction, not only the effects of this intervention but also its implementation must be described in a way that can be replicated.^4^^,^^14^ Although many cooking classes have been implemented in Japan by different bodies with the aim of reducing salt intake,^7^^,^^8^ details concerning the development, content, and implementation of these classes have never been reported, even with clinical trials.^11^^,^^12^

In this study, the authors report a cooking class package aiming to reduce salt in Japanese home cooking. The research team constructed a cooking class and fully described the development and implementation of this class in detail to enable other groups to replicate the approach. In addition, they assessed the impact of this class on salt reduction using data from another clinical trial,^15^ in which this cooking class was offered to a subset of the participants.

## METHODS

**Figure 1:**
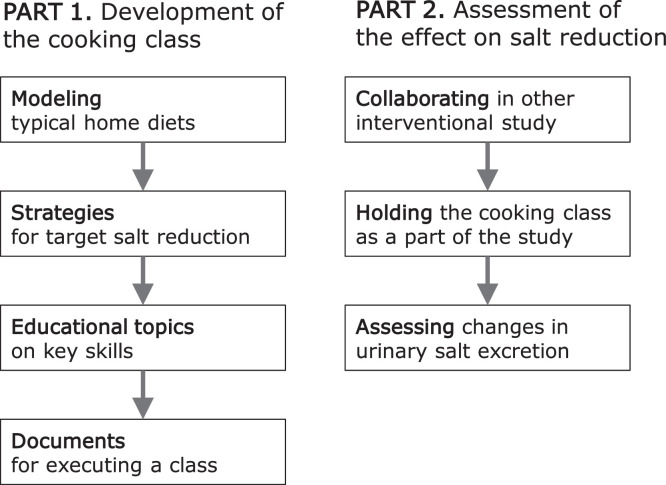
The procedure for developing the cooking class and assessing its effectiveness in this study.

This study consisted of 2 parts: development and assessment (Figure 1).

### Part 1: Development of a Cooking Class for Salt Reduction

A research team consisting of a registered dietitian, a general physician, and a nephrologist was formed to develop a cooking class for salt reduction. The team defined the following requirements for a low-salt cooking class: (1) the amount of salt in the diet is ≤2 g (being ≤1/3 of the recommended daily limit of 6 g for hypertensive patients)^16^; (2) the class content can be integrated into home cooking and is easy to sustain; (3) the class is suitable even for participants without any particular cooking skills or experience; and (4) the class content can be changed according to the region, season, and preferences of participants.

To meet the requirements mentioned earlier, the research team chose to modify and combine traditional salt-reduction instructional methods rather than develop new techniques. To ensure that the class content was modifiable, a step-by-step developmental approach was used, as shown in Table 1. First, the research team modeled the composition of the dishes and the empirically known amount of salt in each dish for traditional Japanese cuisines. The team then developed a combination of strategies to achieve the target salt content without compromising nutritional balance and then established specific educational topics to achieve these goals. Finally, the dietitian on the research team organized these educational elements into a menu and created recipes for the cooking class by referencing the Standard Tables of Food Composition in Japan.^17^ The class length was set at 2 hours. A document was prepared describing the menu, ingredients, ordering, recipes, and how to proceed to enable dietitians who were not part of the research team prepare and host the class.

**Table 1:** Framework of Developing the Cooking Class

Structure of the cooking class in this study					Traditional meals	
Strategy	Educational elements	Menu presented in this study	Salt (g)		Dish category	Salt (g)
1. Restrict the total amount of salt in the main dish	1a. A reliable and easy approach, i.e. determining the total amount of seasonings in the dish and putting it in a pot	Teriyaki^a^ pork	1.0	**←**	Main dish	1.5–3.0
2. Maintain good-tasting meal without needing salty dishes	2a. Excluding salty miso soup and pickles 2b. Adding flavor and aroma to the staple food	Rice cooked with shirasu^b^, perilla leaf, and sesame	0.3	**←**	Staple food Miso soup Salty pickles	0 1.5 0.5–1.5
3. Balance nutrition with low-salt side dishes	3a. Achieving a satisfactory flavor (with vinegar, spices, and dashi) to avoid using salty seasonings at the table 3b. Using vegetables, potatoes, beans, seaweeds, fruits, and dairy products	Carrot salad with mayonnaise Boiled spinach Sweet potatoes simmered in sugar	0.2 0.4 0.0	**←**	Side dish	0.5–1.0
		Total	1.9		Total	4.0–7.0

a Teriyaki, grilling with soy sauce and sugar.

b Shirasu, sardine fry boiled in salty water.

### Part 2: Assessment of the Effect of Salt Reduction

The research team assessed the salt reduction achieved by the cooking class as a secondary analysis of another clinical trial. That clinical trial^15^ investigated the effect of self-monitoring of urinary salt excretion on salt reduction and involved healthy individuals aged ≥20 years from 5 local communities or workplaces in Fukushima prefecture, Japan. It included an 8-week program: half of the participants who were randomly assigned independent of the site self-monitored their salt excretion over the first 4 weeks, and the other half self-monitored over the next 4 weeks. All participants provided an early morning first urine sample at the start (Week 0) and the end (Week 8) of the program.

The present cooking class had been offered to the clinical trial participants in 3 of the 5 sites, independent of the trial allocation, and took place once, in the middle of the program (Week 4). Those 3 sites corresponded simply because facilities and venues for the cooking class were available, whereas the 2 remaining sites did not. The study periods at the 3 and 2 sites were September 2016–March 2017 and November 2016–May 2017, respectively. This secondary analysis was conducted in a nonrandomized design: the participants who had been invited to the cooking class served as the cooking class group, whereas those who had not been invited served as the control group (Table 2).

**Table 2:** Schedule of This Study

Time	Schedule	Group in analysis	
		Cooking class group	Control group
Week 0	Baseline measurement^a^	✓	✓
Week 4	Cooking class	✓	
Week 8	Postprogram measurement^a^	✓	✓

a Participants provided a spot urine sample for the estimation of salt intake.

The outcome was the change in estimated daily salt intake (ESI) (g/day) before and after the program (ΔESI). The ESI was derived from the urine sample using a formula recommended by the Japanese Society of Hypertension,^18^ which is a simple method for obtaining a valid approximation of the 24-hour urinary sodium excretion^19^: 24-hour salt excretion (g/day) = 0.0585 × 21.98 × ([NaS/CrS] × Pr.UCr24) ^ 0.392, where NaS and CrS respectively stand for the sodium and creatinine concentrations in spot urine (mEq/L), and Pr.UCr24 is the estimated 24-hour urinary creatinine excretion (mg/day), with Pr.UCr24= −2.04 × age + 14.89 × body weight (kg) + 16.14 × height (cm) – 2,244.45.

The source clinical trial accepted multiple participants from a single household. Because members of the same household may have the same diet, the research team analyzed 1 participant per household; if more than 1 person participated from 1 household, women were included, and if >1 woman participated, the oldest person was included. The research team included data from all available participants in the analysis rather than formal sample-size calculations.

### Statistical Analysis

Continuous and categorical variables were summarized as the mean (SD) and percentage, respectively. A paired *t*-test was used to test for differences before and after the intervention for continuous variables.

To evaluate the impact of the cooking class, a linear regression model was used, with ΔESI as the dependent variable and participation in the cooking class as the independent variable. The covariates were age (≤65 years), sex, BMI, presence of hypertension, presence of chronic kidney disease, antihypertensive medication, and allocation in the clinical trial. Because allocation in the source clinical trial and having a lifestyle disease as well might have changed participants’ motivation and thus influenced the impact of the cooking class, each interaction of the trial allocation and hypertension with ΔESI was also examined using the same model with an interaction term added. Results were expressed as point estimates of the beta coefficients (corresponding to the difference in ΔESI between the groups) and 95% CIs. Stata 14 (StataCorp, College Station, TX) was used for the statistical analyses.

This secondary analysis was approved by the Ethical Review Committee of Notre Dame Seishin University (30.9.2019). The source clinical trial^15^ was conducted according to the guidelines laid down in the Declaration of Helsinki, and all procedures involving human subjects were approved by the Ethics Committee of Fukushima Medical University (2780). Written informed consent was obtained from all subjects. The trial was registered in the University Hospital Medical Information Network Clinical Trial Registry as UMIN000018870 (https://www.umin.ac.jp/ctr/index.htm).

## RESULTS

### Part 1: Development of a Cooking Class for Salt Reduction

An overview of the cooking class developed in this study is shown in Table 1.

#### Analysis of home cooking

In Japanese food culture, there is a traditional meal pattern that combines plain rice with miso soup and pickles. This is called ichiju-sansai (literally 1 soup and 3 dishes).^20^ The research team first made a model of a typical home cooking pattern (Table 1, right end). It is well known that in the Japanese diet, high-salt soup and pickles are popular components^8^^,^^21^ because they facilitate the consumption of unsalted rice as the staple food. Still, the largest source of salt in the meal is the main dish, including meat or fish.^22^ The research team therefore concluded that to achieve the target salt content, the main dish needed to contain a limited amount of salt, and at the same time, the habit of enjoying miso soup and pickles needed to be curtailed. However, miso soup and pickles also help to balance nutrition in the Japanese diet because this combination contains tofu, vegetables, seaweed, and potatoes.^8^ Efforts to maintain nutritional balance would thus need to be implemented if miso soup and pickles were excluded.

#### Establishing strategies and educational elements

In designing the cooking class, the following 3 strategies were established on the basis of the findings mentioned earlier: (1) determine the amount of salt in the main dish, (2) exclude salty dishes while maintaining the taste of a meal, and (3) add low- or no-salt side dishes to balance nutrition (Table 1, first column). The study team kept the menu and recipes as simple as possible for 2 reasons: to help participants understand the educational elements and to simplify the preparation of the class.

The educational elements defined according to these strategies are shown in the second column of Table 1. For Strategy 1, to ensure that the salt content of the main dish could be reliably controlled, the study team proposed a simple procedure to determine the total amount of salt contained in the dish (1a). Specifically, the study team used soy sauce as a single source of salt and instructed the participants to add a total of 5 ml of soy sauce per person (about 1 g of salt equivalent), determined using a measuring spoon, to the cooking pot. This procedure allows a low-salt dish to be prepared without fail, regardless of the cooking method (boiling, baking, frying, for instance) or ingredients (meat, fish, and others). For Strategy 2, the study team eliminated miso soup and pickles from the meal (2a) and replaced plain rice with staple foods with notable flavors and aromas to compensate for the loss of saltiness (2b). For Strategy 3, the study team strengthened the flavor of the side dishes using vinegar, spices, or dashi (3a)^8^^,^^23^ and emphasized that vegetables should be included as side dishes to maintain nutritional balance (3b).

The class was designed to take place in a laboratory with cooking facilities, where the participants could measure and use seasonings themselves. The participants were planned to learn a goal of 2 g salt per meal and its rationale, how to achieve it as shown in Table 1, and to have a cooking practice along with a tutor demonstration. In addition, time was set aside to allow participants to taste the dishes they had just cooked, helping them understand the relationship between the actual salt content and the taste of a dish.

#### Implementation of cooking classes

The menu developed and used in this class is shown in the third column of Table 1. The salt content was under the target of 2 g/meal, and all of the educational components were met. A detailed description of the approach developed to prepare and execute this cooking class is shown in Appendix Figure A (available online). Using this document, 2 experienced dietitians working in the local government who were not involved in the development of the cooking class were asked to run the class. A total of 15–20 participants were successfully instructed at each of the 3 sites by either 1 of the 2 dietitians.

### Part 2: Assessment of the Effect of Salt Reduction

#### Subjects

**Figure 2:**
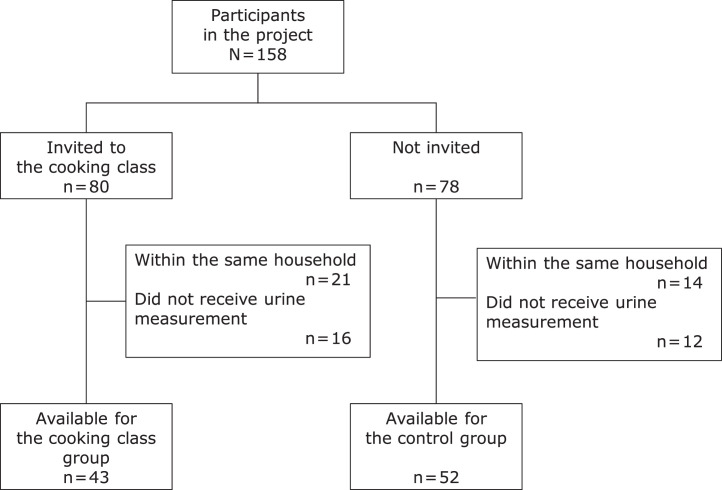
Flow diagram of the subjects for analysis.

Among 158 participants in the clinical trial, 80 were invited to the cooking class, and 78 were not. After excluding subjects within the same household and subjects who did not complete the urinary sodium measurement, 43 and 52 subjects were analyzed for the cooking class group and control group, respectively (Figure 2). The characteristics of subjects in the 2 groups are shown in Table 3. The cooking class group was older, were more female, and had more participants with hypertension and chronic kidney disease. There was no apparent difference in the proportion of clinical trial allocation between the 2 groups.

**Table 3:** Characteristics of Subjects in the Control and Cooking Class Groups

Characteristic	Cooking class group		Control group	
	*n*=43		*n*=52	
Age (years)	67.4	(7.5)	62.4	(10.7)
Female	40	(93.0%)	42	(80.8%)
Hypertension	25	(58.1%)	16	(30.8%)
Chronic kidney disease	4	(9.3%)	2	(3.9%)
BMI (kg/m^2^)	24.0	(3.2)	23.7	(3.8)
Antihypertensive medication	24	(55.8%)	13	(25.0%)
Assigned to the first half of the CT^a^	23	(53.5%)	26	(50.0%)
Urinary salt excretion (g/day)	9.3	(1.9)	9.0	(2.0)

*Note:* Data are presented as mean (SD) and number (proportion).

a In the source CT, the intervention period was randomized to a first half of 4 weeks and a second half of 4 weeks. See text for details. CT, clinical trial.

#### Salt intake

**Figure 3:**
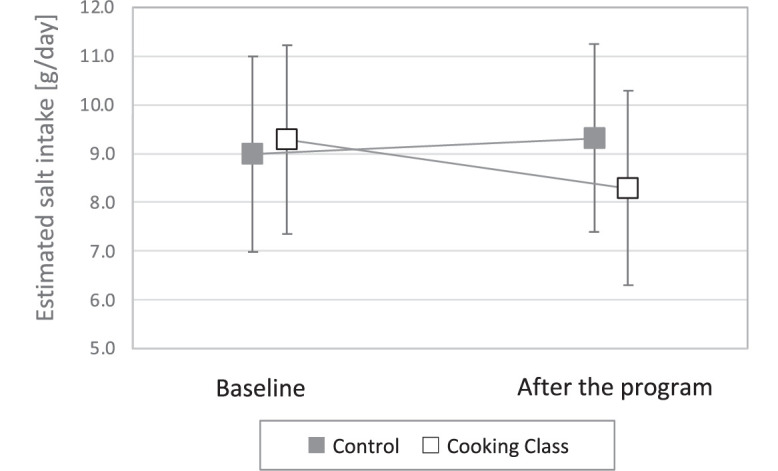
Changes in estimated salt intake (mean and SD) in the control and cooking class groups.

Baseline ESI and postprogram ESI were 9.1 g/day (SD=1.9) and 9.4 (SD=1.9), respectively, in the control group (paired-*t* test *p*=0.18) and 9.2 (SD=2.0) and 8.2 (SD=2.0), respectively, in the cooking class group (*p*=0.0005) (Figure 3). In the regression analysis, the salt reduction was significantly greater in the cooking class group than in the control group (adjusted difference in ΔESI= −1.38 g, 95% CI= −2.19, −0.57 g, *p*=0.001) (Table 4). No other covariates were significantly associated with ΔESI.

**Table 4:** Association of the Cooking Class and Other Covariables With ΔESI

Variables	b-coefficient (95% CI) (g/day)		*p*-value
Cooking class	−1.38	(−2.19, −0.57)	0.001
Group of clinical trial	−0.47	(−1.22, 0.29)	0.225
Age ≥65 years	−0.43	(−1.13, 0.27)	0.229
Sex, female	0.03	(−1.41, 1.46)	0.971
BMI (/kg/m^2^)	−0.04	(−0.16, 0.08)	0.528
Hypertension	−0.62	(−1.70, 0.46)	0.260
Chronic kidney disease	0.31	(−0.63, 1.24)	0.518
Antihypertensive medication	0.91	(−0.20, 2.02)	0.108

ΔESI, Changes in estimated daily salt intake before and after the program.

#### Interaction

The model with the interaction of trial allocation added gave an adjusted difference in ΔESI of −1.05 g (95% CI= −2.10, 0.01 g, *p*=0.050), with a *p*-value for the interaction of 0.46. With the model examining the interaction of hypertension, the adjusted difference in ΔESI was −1.53 g (95% CI= −2.24, −0.61 g, *p*=0.001), with a *p*-value for the interaction of 0.51. Neither of these 2 factors showed interaction with the effect of the cooking class.

## DISCUSSION

In this study, the research team developed a new cooking class on the basis of the previously reported low-salt cooking classes with restructuring to expand their logic and flexibility. They first designed the cooking class by analyzing traditional home diets and structuring effective means of salt reduction. Next, they simplified the hands-on activities so that the participants could focus on their goals and practice methods for salt reduction. When this cooking class was offered to local residents, salt intake after the class was 1.38 g/day lower than in the control group, confirming the salt-reduction effect. To the research team's knowledge, this study was the first to detail the development and content of a cooking class that can be replicated.

Several prominent features of this study warrant mention. First, the cooking class was developed in consultation with medical experts on the research team. By involving multidisciplinary professions, the discussion was not biased toward cooking techniques but always focused on the achievement of salt reduction in residents. Second, the step-by-step approach of defining and breaking down the elements of education to be included in the cooking class made it possible to describe in detail the actual process of developing the cooking class. In this way, cooking classes with similar salt-reduction efficacy can be implemented in different regions, for different populations, and with different menus and recipes. Third, the details concerning the cooking class were described to allow experienced dietitians to reproduce it. Readers may also implement a similar cooking class on their own by referring to this report. Finally, in the assessment of the impact, the research team included the salt intake as determined by a urine test as the outcome rather than a self-assessment by the participants to ensure objectivity. The effect size of 1.38 g/day in this study was comparable with that in previous studies on low-salt Japanese cooking classes (0.9–1.2 g/day).^11^^,^^12^ No modifications to the effect were evident on the basis of the presence of hypertension or the source trial allocation.

Altering an individual's habit of consuming salty food and continuing efforts to reduce salt at home require not only knowledge and motivation about salt reduction but also an understanding of cooking techniques for salt reduction that do not spoil the taste of a meal. Although each policy and technique for low-salt cooking employed in this study was common, supplying consumers with specific suggestions on how and to what extent these approaches should be combined rather than the applications of novel techniques was essential for successfully achieving salt reduction. For the menu and recipes, the research team dared to use common ingredients and basic cooking methods, allowing many variations to be applied, depending on the region, season, and features of participants. In addition, the team presented sample cooking methods that could be applied at home, allowing participants to implement what they learned on a daily basis, forming new, personalized habits by making their own modifications. The reduction of salt in home cooking should have a consequent impact on other members of the household as well. Indeed, an RCT showed that salt-reduction education for housewives subsequently reduced salt intake among other adult family members.^12^ Furthermore, the salt environment in which infants are nurtured reportedly influences their blood pressure 15 years later.^24^ Therefore, the salt-reduction cooking class developed in this study may influence the future salt reduction of not only the individual preparing the meal but also their family.

### Limitations

Several limitations associated with this study warrant mention. First, this cooking class was developed and conducted in a single area of Japan. However, reports from INTERMAP^25^ show relatively small regional differences in the sources of dietary salt intake among Japanese. Second, this was a nonrandomized study. Although the regression model containing some confounders showed no associations with the covariates and interactions, the possibility of unmeasured confounding distorting the results remains. Third, the subjects in this study were participating in a clinical trial on salt reduction and may thus have been in a motivated state. In addition, the research team did not attempt to distinguish the mechanism by which the salt reduction was achieved—whether by participants being further motivated by the cooking class or because they learned cooking skills or both. In any case, the salt-reducing behaviors were improved as a result of participating in this cooking class. Fourth, the effects of repeating the intervention and the long-term effects are unknown. Previous studies have shown a retained effect of salt-reduction education at 3 months^26^ and from 9 to 18 months.^27^ This study, which involved not only the instillation of knowledge but also the accumulation of experience, may have a lasting impact and should thus be examined in further detail in the future.

Consumer education efforts for salt reduction at home may have not given specifics on what to do and to what extent, leaving it up to the consumer's own impressions. Contrary, a cooking class can provide consumers with how to construct a salt-reduction plan and how and to what extent this plan should be implemented to achieve a goal. In this study, the participants learned to assemble salt-reduction behaviors to reach the 2 g salt per meal and implement them. Further studies reporting specific contents of the intervention along with its magnitude of the effects are warranted to establish cooking classes as an evidence-based tool for reducing salt in home cooking.

## CONCLUSIONS

In conclusion, the research team developed a cooking class to reduce salt in Japanese home cooking and reported the development and contents of the class in a reproducible manner. The cooking class was successfully implemented among local residents and shown to have a significant impact on salt reduction.

## CRediT authorship contribution statement

**Miyuki Imamoto:** Conceptualization, Formal analysis, Investigation, Writing – original draft. **Toshihiko Takada:** Investigation, Resources, Writing – review & editing. **Sho Sasaki:** Investigation, Writing – review & editing. **Yoshihiro Onishi:** Writing – original draft, Project administration.

## Appendix Supplementary materials

Image, application 1

Image, application 2

## Footnotes

Supplementary material associated with this article can be found in the online version at doi:10.1016/j.focus.2024.100227.
