# Supplementary material for: Development of a Culinary Intervention (Cooking Class) for Salt Reduction in Japanese Home Cooking: Strategies and Assessment
Source: AJPM Focus. 2024 Mar 13;3(3):100227. doi: 10.1016/j.focus.2024.100227 (PMC11081799; doi:10.1016/j.focus.2024.100227)
Supplement: Supplementary file 1 [file mmc1.pdf]

Element	Staple food	Main dish	Side dishes		
Menu	"Shirasu" rice	"Teriyaki" pork	Carrot salad with mayonnaise	Boiled spinach	Sweet potatoes simmered in sugar
Demonstration/ explanation	1. How to develop a menu without miso soup or pickles<br>2. Understanding nutrients in small fish and seedpods<br>3. How to reduce salt using aromatic vegetables and saltiness of foods	1. Measuring seasonings with a measuring spoon<br>2. Setting the total quantity of salt put in the pot as the product of the per-dish salt content and the number of people	1. How to reduce salt using vinegar and spices<br>2. Nutrients in vegetables and the advisable amount to eat<br>3. Cutting vegetables	1. How to make "dashi" and use it to reduce salt.<br>2. Nutrients in vegetables and the advisable amount to eat<br>3. Boiling vegetables	1. Salt-free side dish recipes and the effect of adding flavor to the surface<br>2. Nutrients in potatoes and the advisable amount to eat<br>3. Cutting and boiling potatoes

	ingredients	1 person	6 servings	ingredients	1 person	6 servings	ingredients	1 person	6 servings	ingredients	1 person	6 servings	ingredients	1 person	6 servings
Foodstuff/ Quantity (for 6 people)	Rice	65 g	390 g	Thin cut of pork	70 g	6 sheets	Carrot	40 g	240 g	Spinach	70 g	420 g	Sweet potatoes	40 g	240 g
	Water	95 g	570 ml	Ginger	1 g	6 g	Asparagus	15 g	6 piaces	"Dashi"	15 g	90 ml	Sugar	3 g	2 tbsp
	Dried "shirasu"	5 g	3 tbsp	Oil	2 g	1 tbsp	Mayonnaise	6 g	3 tbsp	Water	50 g	300 ml			
	Sesame seeds	2 g	1 tbsp	Sugar	3 g	2 tbsp	Vinegar	2.5 g	1 tbsp	"Katsuo-bushi"	1 g	6 g			
	Perilla leaf pieces "shiso"	1 g	6 pices	Soy sauce	6 g	2 tbsp	Whole mustard	3 g	1.5 tbsp	Soy sauce	2.5 ml / 1P	1/2 tsp /1P			
				Japanese sake	5 g	2 tbsp	Pepper	0.01 g	a bit						
				"Mirin"	1 g	1 tsp	Lettuce	10 g	6 pieces						

Time table	"Shirasu" rice	"Teriyaki" pork	Carrot salad with mayonnaise	Boiled spinach	Sweet potatoes simmered in sugar
Cooking 10:30	Cook rice.		Cut the carrots.	Boil the water in a pot.	Cut the sweet potatoes
10:40			Cut the asparagus.	Boil the spinach and squeeze it. in a small pot.	Boil the sweet potatoes
10:50		Heat a pan and add oil and ginger. Grill the pork on both sides.			Drain the water in a colander Add water again and boil
11:00		Wipe the oil from the meat with paper. Add seasoning to the pan.	Boil the asparagus and carrot.		Sprinkle with sugar when
11:10	Cut perilla leaves.	Boil down until water disappears.			
11:20	Mix the ingredients into the rice. Serve and garnish with perilla leaves.	Serve.	Season boiled vegetables. Serve with lettuce.	Soak the spinach in the "dashi" and squeeze it.	Serve.
Trying dishes 11:30					
Clean up 12:30					

Setting of the cooking class for 36 people

1. Cooking room: 6 tables + instructor's table
2. Cooking class staff: 6 (2 dietitians, 4 assistants)

Appendix Figure A. Manual for implementation of the cooking class for salt reduction
